# Smiling makes you look older, even when you wear a mask: the effect of face masks on age perception

**DOI:** 10.1186/s41235-022-00432-3

**Published:** 2022-09-06

**Authors:** Tzvi Ganel, Melvyn A. Goodale

**Affiliations:** 1 Psychology Department, Ben-Gurion University of the Negev, 8410500 Beer-Sheva, Israel; 2 Department of Psychology and the Western Institute for Neuroscience, The University of Western Ontario, London, ON N6A 5B7 Canada

**Keywords:** Face perception, Masked faces, Age evaluations, Facial expression, Smiling

## Abstract

The widespread use of face masks in the era of the Covid-19 pandemic has promoted research on their effect on the perception and recognition of faces. There is growing evidence that masks hinder the recognition of identity and expression, as well as the interpretation of speech from facial cues. It is less clear whether and in what manner masks affect the perception of age from facial cues. Recent research has emphasized the role of the upper region of the face, a part not covered by a mask, in the evaluation of age. For example, smile-related wrinkles in the region of the eyes make smiling faces appear older than neutral faces of the same individuals (the aging effect of smiling, AES). In two experiments, we tested the effect of face masks on age evaluations of neutral and smiling faces in a range of different age groups from 20 to 80 years. The results showed that smiling faces were perceived as older than neutral faces even when individuals were wearing a face mask—and there was no effect of masks on bias in age evaluations. Additional analyses showed reduced accuracy in age evaluations for smiling compared to neutral faces and for masked compared to unmasked faces. The results converge on previous studies emphasizing the importance of the upper region of the face in evaluations of age.

## Significance statement

In this manuscript, we provide a comprehensive investigation of the effect of masks on different aspects of age evaluations. Within this context, we looked at the effect of masks on age evaluations of neutral and smiling faces. We found that wearing a face mask does not diminish the well-established effect of smiling on age perception: the fact that when people smile, they look older. In addition, we showed that contrary to previous suggestions, masks do not make people appear to be younger or older. The only difference in age evaluations between masked and unmasked faces was a moderate decrease in accuracy for age evaluations of masked faces. The findings confirm that the perception of age is driven largely by the upper part of the face and that the wrinkling of the eyes that occurs when people smile is responsible for the bias in age perception. These results provide timely insights on the effect of masks on face perception and on the processes that underlie the perception of facial age.

## Introduction

Extracting accurate information about the age of an individual allows for more effective social interactions. It is not surprising, therefore, that among the different features that people can readily extract from a person’s face, age is considered primary (George & Hole, 1998). To evaluate the age of someone’s face effectively, observers must take into account a wide range of age cues, including the overall shape of the face, the person’s hairstyle and hair colour, as well as the prominence of wrinkles and skin pigmentation (Lai et al., 2013; Voelkle et al., 2012). Although these age cues allow humans to achieve impressive accuracy in evaluating age, performance is still imprecise, leaving a large space for errors and biases.

One such bias, described in a series of recent studies conducted in our lab, is the aging effect of smiling or AES: the fact that when a person smiles, they are perceived as older than when they maintain a neutral expression (Ganel, 2015; Ganel & Goodale, 2018, 2021). This bias has been shown to be a consequence of the failure of the observer to discount temporary information from smiling-induced wrinkling in the upper part of the face and, in particular, in the region of the eyes (Ganel, 2015). The perception of smiling faces as older is unintuitive, going against the common belief that smiling makes people look younger, not older (Ganel & Goodale, 2018). Recently, the AES has been extended for own- and other-race faces (Yoshimura et al., 2021), and for own-race faces of different age groups (Ganel & Goodale, 2021). In particular, the AES was found for male and female faces in young people and for male faces in middle-aged people. Perhaps not surprisingly, no AES was found for faces of old adults, probably because they already have many facial wrinkles and other facial cues that mark them as older (Ganel & Goodale, 2021).

The widespread usage of face masks in the era of the COVID-19 pandemic has created an opportunity for timely research on the effect of masks on different aspects of face perception and recognition. So far, research has shown that wearing a face mask can lead to decreased or even abnormal performance in key aspects of face processing. Such aspects include the identification of unfamiliar and familiar faces (Carragher & Hancock, 2020; Freud et al., 2020; Noyes et al., 2021), speech perception (Magee et al., 2020; Truong & Weber, 2021; Truong et al., 2021), and the processing of facial emotions both in children and in adults (Carbon, 2020; Carbon & Serrano, 2021; Gori et al., 2021; Grundmann et al., 2021; Marini et al., 2021; Ruba & Pollak, 2020). It is less clear, however, whether and in which manner masks affect the perception of age from a person’s face. Given the key role of the upper face region, which is typically not covered by masks, in age perception, it might be expected that masks would not lead to directional biases in the evaluation of age. Yet, the scarce research in this domain has not yielded firm conclusions as to the possibility of directional effects of faces masks on age evaluation.

One recent study that looked at the effect of masks on direct age evaluations reported that masked faces of middle-aged adults were perceived as younger than unmasked faces of the same individuals (Nicksic et al., 2021). This study was limited, however, by serious methodological confounds (e.g. unbalanced and limited set of faces, small and biased participant sample, differences in the lighting and colouring of photographs of masked vs. unmasked faces), which make it hard to conclude that face masks lead to such a directional bias in age estimations. More recently, two other studies have also looked at the possibility of directional biases in age estimations due to masks (Lau, 2021; Lau & Huckauf, 2021). The results were inconsistent: while Lau (2021) found that masked faces are perceived as younger than unmasked faces, the results of Lau and Huckauf (2021) showed the opposite effect (see also Thorley et al., 2022). These discrepancies could have resulted from item-specific effects due to the relatively small sample of faces used in each study (only 8 different exemplars were presented in each of the studies). Therefore, the possibility of mask-induced biases in age perception warrants a more comprehensive investigation across a larger set of face exemplars and across a large age range, which was the case in the current study.

**Fig. 1:**
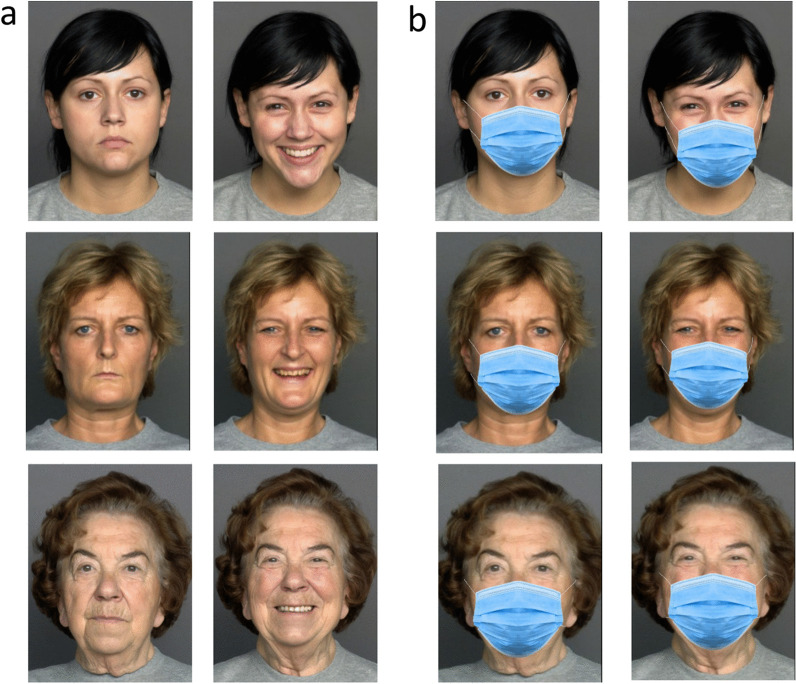
Sample of the stimuli used in Experiments 1 and 2. **a** Unmasked neutral and smiling faces of young adults, middle-aged adults, and old adults. **b** Masked versions of the same faces. The unmasked faces were adapted from Ebner, N. C., Riediger, M., & Lindenberger, U. (2010). FACES—A database of facial expressions in young, middle-aged, and older women and men: Development and validation. Behavior Research Methods, 42, 351–362. 10.3758/BRM.42.1.351, all rights reserved

In the current study, we carried out a comprehensive investigation of the effect of face masks on age evaluation. To do this, we used a modified version of a large face database used in our most recent study to investigate the effect of smiling on age perception. The database includes photographs of 240 female and male faces from different age groups (ranging in age between 20 and 80 years). Each person was photographed in a smiling and in a neutral expression. A masked version of the entire set was created by graphically adding face masks to the photographs (see Fig. 1). Participants were asked to evaluate the age (in years) of each photograph in the set. This design allowed us to test different aspects related to the effect of masks on age evaluations.

In particular, we were able to test the presence of AES in masked faces and unmasked faces within the same design. Given that the recognition of facial expression is impaired for masked faces (Grundmann et al., 2021), it is possible that viewers would have difficulty discounting the presence of wrinkling to the presence of a smile. It is therefore predicted that to the extent that the AES is mediated by such explicit awareness of smiling, the AES would be present, or would be even stronger for masked compared to unmasked faces. Our design also allowed us to test for possible biases due to masks on average age evaluations of faces in a large set of face exemplars and across different age groups of faces. Lastly, we were also able to test the effect of masks and the effect of smiling on the accuracy in face evaluations of male and female faces in different age groups.

## Experiments 1 and 2

In Experiments 1 and 2, participants were presented with a series of neutral and smiling masked or unmasked faces. Participants were asked to perform age evaluations for each of the 240 unique identities in the set of faces presented to them. In Experiment 1, we focused on the AES in masked faces. The unmasked face condition served as baseline and its design was similar to the one used in our recent study (Ganel & Goodale, 2021). Two different groups of observers participated in the masked and unmasked conditions. In Experiment 2, we focused more closely on possible effects of face masks on perceived age. The design was similar to the one used in Experiment 1, but now, face masking was manipulated as a within-subject variable. Therefore, participants in Experiment 2 performed age evaluation for a series of 240 masked and unmasked faces with neutral and smiling expressions. The presentation order in the two experiments was counterbalanced so that none of the unique identities of the 240 people in the set were repeated more than once for each participant (see Ganel & Goodale, 2021).

## Method

### Participants

All experiments were performed online. Participants were recruited from the Prolific online participant pool. Eight-six participants took part in Experiment 1 (46 in the masked condition and 40 in the unmasked condition, 41 females, mean age = 25.9 years, SD = 8.3 years) and 80 different participants (52 females, mean age = 24.53 years, SD = 7.3) participated in Experiment 2. Sample sizes in Experiment 1 were based on those used in our previous study of the AES (Ganel & Goodale, 2018). In Experiment 2, which was designed to directly test the effect of masks on age evaluations, we used a sample size similar to that used in Experiment 1, but now in a within-subject design. The experimental protocol was approved by the ethics committee of the Department of Psychology in Ben-Gurion University of the Negev. The study adhered to the ethical standards of the Declaration of Helsinki. All participants signed an informed consent form prior to their participation in the experiment. The data of 6 participants (2 from Experiment 1) were removed from the analysis due to a large error in age evaluations (larger than 3 standard deviations above the mean).

### Design and materials

The face database we used was based on an unmasked set we used in a recent study (for full description, see Ganel & Goodale, 2021). A masked version of the set was created using the Face Mask Photo Editor app. Each set (of masked and unmasked faces) contained photographs of 120 women and 120 men, each with neutral or smiling expressions. The photographs were divided into 3 age groups: young adults (20–39 years), middle-aged adults (40–59), and old adults (60–80 years). The average age of the young adult group was 24.94 years old (24.93 for female faces, 24.95 for male faces). The average age of the middle-aged adult group was 49.1 years old (49.83 for females, 48.38 for males). The average age of the old adult group was 71.39 years old (71.3 for females, 71.48 for males). Stimuli were cropped to the dimensions of about 375X500 pixels. Examples of the photographs are presented in Fig. 1.

### Experimental procedure

The procedures used in Experiments 1 and 2 were similar, except that Experiment 1 used a between-subjects design and Experiment 2 a within-subject design (more sensitive to the effect of face masks on age evaluations). We designed the experiments so that each participant was presented with only one photograph of each specific identity (see Ganel, 2015; Ganel & Goodale, 2018). The design of Experiment 1 was similar to that used in our previous study (Ganel & Goodale, 2021), but now was applied to faces with masks as well. The stimulus set in Experiment 1 was divided into two equal subsets of 120 photographs (sets A and B) within each masking condition. For half of the participants, the faces in set A were smiling and those in set B displayed a neutral expression and for the other half, set B was smiling and set A had a neutral expression. The stimulus set in Experiment 2 was composed of the same masked and unmasked faces used in Experiment 1. The stimulus set was now divided into 4 equal subsets (A–D). Each participant was assigned to one of four combinations of expression (smiling, neutral) and masking (masked, unmasked) for each set. The faces from the different subsets were presented in a random order. Each face was presented on the screen until a response was made. Participants typed their response in years, which appeared below the target photo, and then pressed the "Continue" button to proceed to the next trial. Responses that were not 2-digit numbers were excluded from the analysis (less than 1% of the total responses).

### Analysis

Analyses were conducted using JAMOVI 2 and Statistica 13.5. The main dependent variables were the average perceived age and the mean accuracy score in each of combination of age group X gender X expression X masking condition. For each participant, accuracy scores were computed by calculating the average absolute difference between the perceived and real age of each of the faces in each experimental combination. A mixed ANOVA design with the gender of the photographed person, his/her expression (smiling, neutral), and age group as the within-subject independent variables and the presentation format (masked, unmasked) as a between-subjects independent variable was used to analyse the data in Experiment 1. A repeated-measures ANOVA design with presentation format, gender, expression, and age group was used to analyse the data in Experiment 2. For the post hoc comparisons of the effect of perceived age (bias in age perception) in each experimental combination, we applied Bonferroni-corrected *α* = 0.05 ÷ 12 = 0.0042. For the ANOVA analyses, Greenhouse–Geisser corrections were used for effects that violated the sphericity assumption.

## Results

### Experiment 1: between-subjects manipulation of face masks

**Fig. 2:**
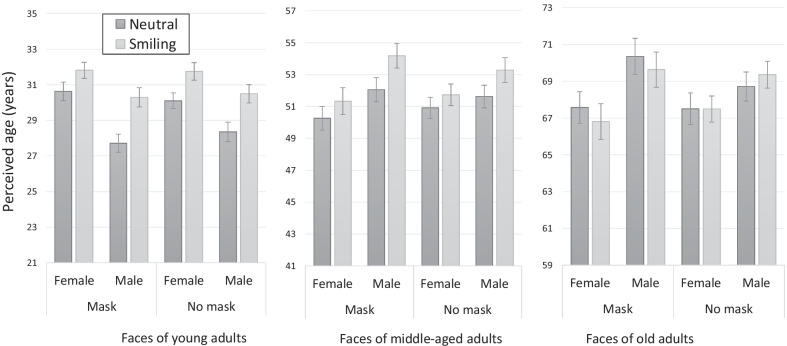
The aging effect of smiling (AES) for masked and unmasked faces in Experiment 1. For unmasked faces, AES was found for male and for female faces in young adults, and for male faces in middle-aged adults. A similar pattern of results was found for masked faces, but now AES was also found for middle-aged female faces. Error bars represent standard errors of the mean

The mean perceived ages of masked and unmasked neutral and smiling faces are presented in Fig. 2. As can be seen in the figure, the results of the unmasked condition replicate our previous findings of an aging effect of smiling (AES) for female and male faces in the young adults, and for male faces in the middle-aged adults group. There was no effect of smiling on the age evaluations of the elderly group. The pattern of results for masked faces was similar, but now there was an indication for AES for middle-aged adult females as well.

A mixed ANOVA design with the gender of the photographed person, expression, and age group as the within-subject independent variables and the presentation format (masked, unmasked) as a between-subjects independent variable was used to analyse the data, with perceived age (years) serving as the dependent variable. Preliminary analysis that included the gender of the participants did not show a main effect of gender or interactions with presentation format or expression and therefore, the participant’s gender was not included here or in further analyses.

Main effects were found for age group [*F*(1.33,109.27) = 3313.57, *p* < 0.001, *η*_p_^2^ = 0.98] and for expression [*F*(1,82) = 81.67, *p* < 0.001, *η*_p_^2^ = 0.49], indicating that, overall, smiling faces were perceived as older than neutral faces. The main effect of gender (of the photographed person) was significant with female faces perceived as younger than male faces [*F*(1,82) = 13.8, *p* < 0.001, *η*_p_^2^ = 0.14]. More importantly, there was no effect of mask on perceived age, as indicated by a nonsignificant main effect of format [*F*(1,82) = 0.02, *p* > 0.05]. This result showed that face masks do not produce biases along perceived age across the different conditions.

A significant interaction between gender and expression [*F*(1,82) = 12.43, *p* < 0.001, *η*_p_^2^ = 0.13] indicated larger AES for male compared to female faces (for similar results, see Ganel & Goodale, 2021). A significant interaction between age group and gender [*F*(2,164) = 99.79, *p* < 0.001, *η*_p_^2^ = 0.55], indicated that the effect of gender was different in the three age groups. This interaction was qualified, however, by a three-way interaction with presentation format [*F*(2,164) = 6.42, *p* < 0.001, *η*_p_^2^ = 0.07] and, as described below, was further explored using specific comparisons. A significant interaction was also found between age group and expression [*F*(2,164) = 34.39, *p* < 0.001, *η*_p_^2^ = 0.29], indicating different effects of smiling on perceived age in the different age groups (Ganel & Goodale, 2021). Again, this interaction was qualified by a three-way interaction with format [*F*(2,164) = 3.78, *p* < 0.05, *η*_p_^2^ = 0.04]. The two-way interactions between age group and format [*F*(2,164) < 1, *p* > 0.05], between gender and format [*F*(1,82) = 2.49, *p* > 0.05, *η*_p_^2^ = 0.03], and between expression and format [*F*(1,82) = 1.07, *p* > 0.05, *η*_p_^2^ = 0.01] were not significant. The interactions between gender, expression, and format [*F*(1,82) < 1, *p* > 0.05], between gender, expression, and age group [*F*(2,164) = 1.02, *p* > 0.05, *η*_p_^2^ = 0.01], and the four-way interaction [*F*(2,164) = 1.31, *p* > 0.05, *η*_p_^2^ = 0.01] were not significant as well. To better understand the pattern of results and to test for the presence of AES in the different conditions, we performed planned comparisons between smiling and neutral female and male faces within each age group.

The results of the unmasked condition replicated those of our recent study with unmasked faces (Ganel & Goodale, 2021). Planned comparisons showed an aging effect of smiling (AES) for female [*F*(1,82) = 23.72, *p* < 0.001, *η*_p_^2^ = 0.22] and for male faces [*F*(1,82) = 46.76, *p* < 0.001, *η*_p_^2^ = 0.36] of young adults, but only for male faces of middle-aged adults [*F*(1,82) = 53.69, *p* < 0.001, *η*_p_^2^ = 0.19]. AES was not found for either male or female elderly faces.

A similar pattern of results was found for masked faces. Planned comparisons showed significant AES for female [*F*(1,82) = 14.09, *p* < 0.001, *η*_p_^2^ = 0.15] and male faces [*F*(1,82) = 78.66, *p* < 0.001, *η*_p_^2^ = 0.49] of young adults, and for male faces of middle-aged adults [*F*(1,82) = 35.45, *p* < 0.001, *η*_p_^2^ = 0.30]. Interestingly, the AES was now present for female faces of middle-aged adults [*F*(1,82) = 6.31, *p* < 0.05, *η*_p_^2^ = 0.07]. For old adults, there was an unexpected trend in the opposite direction, with smiling female [*F*(1,82) = 4.22, *p* < 0.05, *η*_p_^2^ = 0.05] and male faces [*F*(1,82) = 4.91, *p* < 0.05, *η*_p_^2^ = 0.06] perceived as younger than neutral faces. Due to the unpredicted small effect sizes, however, these results should be interpreted with caution.

To further test for possible mask-induced biases in age perception, we performed post hoc comparisons of the effect of mask within each combination of gender and expression in each age group. None of the comparisons were significant (all *F*’s < 1). These results coincide with the non-significant main effect of presentation format and suggest that masks do not lead to directional biases in age evaluations.

### Accuracy in age evaluation

To test if masks interfered with the accuracy of age evaluations, we computed accuracy scores by calculating the average absolute difference between the perceived and real age of each of the faces in each combination of age group, gender, and expression. Accuracy scores for the different conditions are shown in Table 1. As can be seen in the table, accuracy decreased with age group and was overall lower for smiling compared to neutral faces (see Ganel & Goodale, 2021; Voelkle et al., 2012).

**Table 1:** Mean accuracy (absolute errors in years) of age evaluations in Experiment 1 (standard deviations in brackets). Note that larger numbers indicate lower accuracy

Age group (of faces)		Young adults		Middle-aged adults		Old adults	
Gender (of faces)		Female	Male	Female	Male	Female	Male
Non-masked faces	Neutral faces	6.62 (2.6)	5.54 (2.2)	7.17 (2.5)	7.29 (2.2)	7.98 (2.9)	7.73 (3)
	Smiling faces	7.73 (2.3)	7.35 (2.4)	8.26 (2.7)	8.13 (3.3)	8.43 (3.2)	7.48 (2.7)
Masked faces	Neutral faces	6.86 (2.4)	5.84 (2.1)	7.43 (2.4)	7.8 (2.5)	7.99 (3.1)	6.78 (2.7)
	Smiling faces	7.95 (2.5)	7.46 (2.5)	7.99 (2.3)	8.95 (3.4)	8.17 (3)	6.97 (2.2)

A mixed ANOVA design with gender, expression, and age group as the within-subject independent variables and with presentation format as a between-subjects independent variable was used to analyse the accuracy data.

A main effect was found for age group [*F*(1.67,136.9) = 4.76, *p* < 0.05, *η*_p_^2^ = 0.05], reflecting higher accuracy in age judgments for young compared to middle-aged and old adults. A main effect was also found for expression [*F*(1,82) = 96.68, *p* < 0.001, *η*_p_^2^ = 0.54], indicating reduced accuracy for smiling compared to neutral faces (Ganel & Goodale, 2021; Voelkle et al., 2012). The main effect of gender was significant [*F*(1,82) = 14.48, *p* < 0.001, *η*_p_^2^ = 0.15], indicating overall reduced accuracy for female faces. This effect was qualified by a significant age group X gender interaction [*F*(1,82) = 12.81, *p* < 0.001, *η*_p_^2^ = 0.14]. In addition, there was a significant interaction between age group and expression [*F*(2,164) = 20.68, *p* < 0.001, *η*_p_^2^ = 0.19], resulting from a reduced effect of expression on accuracy scores in the old age group. As in the main analysis, there was no effect of masks on accuracy, indicated by a non-significant main effect of presentation format [*F*(1,82) = 0.01, *p* > 0.05]. The two-way interactions between age group and format [*F*(2,164) < 1, *p* > 0.05], between gender and format [*F*(1,82) < 1, *p* > 0.05], and between expression and format [*F*(1,82) < 1, *p* > 0.05] were not significant. The interactions between gender, expression, and format [*F*(1,82) = 1.98, *p* > 0.05, *η*_p_^2^ = 0.02], between gender, expression, and age group [*F*(2,164) = 2.62, *p* > 0.05, *η*_p_^2^ = 0.03], between gender, age group and format [*F*(2,164) = 2.95, *p* > 0.05, *η*_p_^2^ = 0.03], and the four-way interaction [*F*(2,164) < 1, *p* > 0.05] were all not significant.

### Response times

Response times were not the main dependent variable in our design (participants were not required to complete their age estimation in a speeded manner). Still, we analysed response times data to account for the possibility of speed-accuracy trade-off in accuracy of age evaluations. Response times were measured from the time of the presentation of the face until participants pressed the “Continue” button after they have completed to type their age evaluation in years. Outliers larger or smaller than 3 standard deviations above or below the mean were excluded from the response times analysis. Mean response times are presented in Table 2. For sake of brevity, we did not include the gender of the face in this analysis.

**Table 2:** Mean response times (in ms) to complete age evaluations of unmasked and masked neutral and smiling faces in the different age groups in Experiments 1 and 2 (standard errors of the mean in brackets)

Age group (of faces)		Young adults		Middle-aged adults		Old adults	
Expression		Neutral	Smiling	Neutral	Smiling	Neutral	Smiling
Experiment 1	Unmasked faces	4555 (272)	4757 (346)	4869 (347)	4825 (291)	4580 (347)	4451 (323)
	Masked faces	5005 (345)	5008 (316)	5354 (342)	5172 (332)	4941 (311)	4972 (324)
Experiment 2	Unmasked faces	4979 (208)	5107 (210)	5262 (197)	5359 (209)	4985 (196)	4847 (179)
	Masked faces	4925 (207)	5264 (241)	5345 (219)	5302 (222)	5163 (221)	4971 (211)

A mixed ANOVA with expression and age group as the within-subject independent variables and with presentation format as a between-subjects independent variable showed a main effect of age group [*F*(2,168) = 12.74, *p* < 0.001, *η*_p_^2^ = 0.13]. This effect resulted from longer response times to evaluate the ages of middle-aged adults faces compared to the other two age groups. The effect of presentation format was not significant [*F*(1,84) < 1], excluding the possibility of a speed-accuracy trade-off. All other effects and interactions were not significant and are not reported for sake of brevity.

The results of Experiment 1 extend our previous findings and show that the AES continues to be present in masked faces. This finding, together with the similar pattern of results found for unmasked and masked faces, suggests that age evaluations rely on visual information from regions of the face that are not covered by masks. It is still possible that the design of Experiment 1, which was focused on the effect of smiling in masked (and unmasked) faces, was not sensitive enough to detect possible effects of masks on age perception. Experiment 2 was designed to resolve this concern using a within-subject manipulation of masking that can provide a more sensitive measure for detecting possible effects of masks on the perception of age of neutral and smiling faces.

### Experiment 2: within-subject manipulation of face masks

**Fig. 3:**
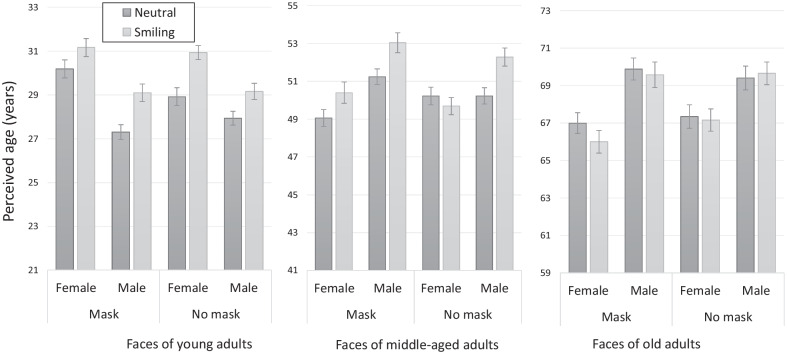
The aging effect of smiling (AES) for masked and unmasked faces in Experiment 2. The results provide close replication of the results in Experiment 1. For masked faces, AES was found in young and middle-aged adults male and female photographs. Error bars represent standard errors of the mean

The mean perceived ages in the different categories are presented in Fig. 3. As can be seen in the figure, the results provide a close replication of the results of Experiment 1. In particular, in the unmasked condition, AES were found for female and male faces for young adults, and for male faces for middle-aged adults. AES was found in these groups and also for middle-aged adult females in the masked condition. There was no AES present in either the unmasked or the masked elderly faces.

A repeated-measures ANOVA with presentation format (masked, unmasked), gender (of the face), expression, and age group as within-subject independent variables was used to analyse the data of the perceived age (in years). As in Experiment 1, main effects were found for age group [*F*(1.34,100.4) = 3486.48, *p* < 0.001, *η*_p_^2^ = 0.98] for expression [*F*(1,75) = 41.88, *p* < 0.001, *η*_p_^2^ = 0.36], and for gender [*F*(1,75) = 32.39, *p* < 0.001, *η*_p_^2^ = 0.3]. As in Experiment 1, the main effect of mask was not significant [*F*(1,75) = 0.29, *p* > 0.05]. Again, this result shows that across all age groups and conditions, perceived age is not biased by the presence of a mask.

As in Experiment 1, there was a significant interaction between gender and expression [*F*(1,75) = 15.26, *p* < 0.001, *η*_p_^2^ = 0.17], with a larger AES for male compared to female faces. Again, the significant interaction between age group and gender [*F*(2,150) = 142.1, *p* < 0.001, *η*_p_^2^ = 0.66] was qualified by a three-way interaction with presentation format (masked vs. unmasked) [*F*(2,150) = 11.23, *p* < 0.001, *η*_p_^2^ = 0.13]. A significant interaction was again found between age group and expression [*F*(2,150) = 20.26, *p* < 0.001, *η*_p_^2^ = 0.21], indicating different AES in the different age groups. This interaction was qualified by a three-way interaction with gender [*F*(2,150) = 4.94, *p* < 0.05, *η*_p_^2^ = 0.06]. The four-way interaction was also significant [*F*(1.65,123.38) = 5.33, *p* < 0.001, *η*_p_^2^ = 0.07]. The two-way interactions between gender and format [*F*(1,75) = 2.17, *p* > 0.05, *η*_p_^2^ = 0.03], between expression and format [*F*(1,75 < 1, *p* > 0.05] and the three-way interactions between gender, expression, and format [*F*(1,75) < 1, *p* > 0.05], between age group, expression, and format [*F*(1.58, 118.16) = 2.05, *p* > 0.05, *η*_p_^2^ = 0.03] were all not significant.

Planned comparisons between smiling and neutral faces were performed to the test the presence of AES in the different conditions. In the unmasked condition, AES was again found for female [*F*(1,75) = 29.57, *p* < 0.001, *η*_p_^2^ = 0.28] and male faces [*F*(1,75) = 15.91, *p* < 0.001, *η*_p_^2^ = 0.16] of young adults, but only for male faces of middle-aged adults [*F*(1,75) = 31.17, *p* < 0.001, *η*_p_^2^ = 0.29]. AES was not found for the faces of elderly male and female individuals. The pattern of results for masked faces was also similar to the one obtained in Experiment 1. A significant AES was found for female [*F*(1,75) = 5.31, *p* < 0.05, *η*_p_^2^ = 0.07] and male faces [*F*(1,75) = 34.23, *p* < 0.001, *η*_p_^2^ = 0.31] of young adults and for female [*F*(1,75) = 6.9, *p* < 0.05, *η*_p_^2^ = 0.08] and male faces [*F*(1,75) = 18.58, *p* < 0.001, *η*_p_^2^ = 0.19] of middle-aged adults. Unlike in Experiment 1, there was no difference between smiling and neutral faces of old adult males [*F*(1,75) < 1, *p* < 0.05]. Similar to the results of Experiment 1, smiling faces of old adult females were perceived as younger than faces with a neutral expression [*F*(1,75) = 8.19, *p* < 0.001, *η*_p_^2^ = 0.09].

As in Experiment 1, we used post hoc comparisons to test for possible mask-induced biases within each combination of gender and expression in each age group. The pattern of results was inconsistent, both in terms of magnitude and direction, and in terms of the statistical significance. Out of the 12 specific comparisons three were significant. Out of these three comparisons, two went in one direction (faces with masks were perceived as slightly younger) and one went in the opposite direction (faces with masks were perceived as slightly older). In particular, masked faces were perceived as significantly older than unmasked faces for young adult neutral females [*F*(1,75) = 9.71, *p* < 0.05, *η*_p_^2^ = 0.11] and for middle-aged adults neutral males [*F*(1,75) = 9.18, *p* < 0.05, *η*_p_^2^ = 0.11]. At the same time, masked faces were perceived as marginally younger for older adult female smiling faces [*F*(1,75) = 8.41, *p* = 0.058, *η*_p_^2^ = 0.1]. This inconsistent pattern of results is in agreement with the nonsignificant main effect of presentation format and show once again that masks do not impose a general (directional) bias on age evaluations.

### Accuracy in age evaluations

Accuracy scores for the different conditions are shown in Table 3. As in Experiment 1, accuracy decreased with age group and was lower for smiling compared to neutral faces. Unlike Experiment 1, however, accuracy in the different age groups was lower for masked compared to unmasked faces.

**Table 3:** Mean accuracy (absolute errors in years) of age evaluations in Experiment 2 (standard deviations in brackets)

Age group (of faces)		Young adults		Middle-aged adults		Old adults	
Gender (of faces)		Female	Male	Female	Male	Female	Male
Non-masked faces	Neutral faces	5.52 (1.9)	5.18 (1.7)	6.37 (2.1)	6.29 (1.8)	7.99 (3.2)	7.03 (2.3)
	Smiling faces	7.18 (2.6)	6.35 (2.2)	6.78 (2.6)	7.05 (2.5)	8.53 (2.7)	7.17 (2.5)
Masked faces	Neutral faces	6.62 (2.4)	5.23 (1.7)	7 (2.3)	7.04 (2.5)	8.2 (2.9)	6.96 (2.6)
	Smiling faces	7.51 (2.6)	6.48 (2.5)	7.66 (2.4)	8.15 (2.7)	8.83 (3.3)	7.15 (2.8)

Note that larger numbers indicate lower accuracy

A repeated-measures ANOVA with presentation format, gender, expression, and age group was used to analyse the accuracy data. The main difference between the accuracy results here and in Experiment 1 was the significant reduction in accuracy for masked faces. This was indicated by a main effect of presentation format [*F*(1,75) = 18.54, *p* < 0.001, *η*_p_^2^ = 0.19]. This main effect was qualified by format X gender interaction [*F*(1,75) = 6.83, *p* < 0.05, *η*_p_^2^ = 0.08], which resulted from smaller effect of format for male compared to female faces. As in Experiment 1, main effects were found for age group [*F*(2,150) = 14.03, *p* < 0.001, *η*_p_^2^ = 0.16], expression [*F*(1,75) = 101.52, *p* < 0.001, *η*_p_^2^ = 0.58, and gender [*F*(1,75) = 28.22, *p* < 0.001, *η*_p_^2^ = 0.27]. The main effect of gender was qualified by significant age group X gender interaction [*F*(1,75) = 24.96, *p* < 0.001, *η*_p_^2^ = 0.25]. As was the case in Experiment 1, the interaction between age group and expression was significant [*F*(2,150) = 10.52, *p* < 0.001, *η*_p_^2^ = 0.12]. The three-way interaction between gender, expression, and age group was also significant, *F*(2,150) = 6.61, *p* < 0.001, *η*_p_^2^ = 0.08]. The two-way interactions between gender and expression [*F*(1,75) = 1.13, *p* > 0.05, *η*_p_^2^ = 0.005], between age group and format [*F*(2,150 = 2.71, *p* > 0.05. *η*_p_^2^ = 0.03], between expression and format [*F*(1,75) = 1.74, *p* > 0.05, *η*_p_^2^ = 0.01] and the three-way interactions between gender, expression, and format [*F*(1,75) < 1, *p* > 0.05], and between age group, expression, and format [*F*(2,150) < 1, *p* > 0.05] were not significant.

### Response times

Mean response times are presented in Table 2. As in Experiment 1, a mixed ANOVA with expression and age group as the within-subject independent variables and with presentation format as a between-subjects independent variable showed a main effect of age group [*F*(1.8,144.9) = 9.62, *p* < 0.001, *η*_p_^2^ = 0.11]. As in Experiment 1, this effect was a consequence of longer response times to evaluate the ages of middle-aged adults faces compared to the other two age groups. The interaction between age group and expression was also significant [*F*(2,158) = 4.71, *p* < 0.01, *η*_p_^2^ = 0.056]. All other effects and interactions were not significant.

## Discussion

The primary purpose of the two experiments in the current study was to look at the effect of face masks on the evaluation of a person’s age. To this purpose, we examined whether masked smiling faces are perceived as older than masked neutral faces (the AES), as is reliably the case for unmasked smiling faces. The results of the two experiments, one using a between-subjects design and the other a within-subject design, converged on the same conclusion: smiling faces, even when they are masked, are perceived as older than their neutral counterparts. Moreover, this result is a strong confirmation of our earlier work showing that the AES is driven by smile-related wrinkles around the eyes.

The results with unmasked faces in the current study provide a robust replication of our previous findings (Ganel & Goodale, 2021). In particular, smiling faces were perceived as older than neutral faces for young female and male adults as well as for middle-aged male adults. AES was not found for old adults, probably due to the wealth of age cues and existing wrinkles in faces of old adults that offset information from smile-related wrinkles in the region of the eyes (Ganel & Goodale, 2021).

The pattern of results for masked faces closely resembled the pattern found for the unmasked faces across the different age groups. We note two differences, however, between the expression of the AES for masked and unmasked faces. First, unlike what happens in the case of unmasked faces, a significant AES was found for masked faces of middle-aged females in Experiments 1 and 2. This stronger AES in the masked condition is expected, given that the smile-related wrinkles in the region of the eyes not covered by the mask are no longer offset by the presence of other wrinkles and facial cues to age in the covered part of the face in the masked condition (for similar results, see Ganel & Goodale, 2021; Ganel, 2015) An unpredicted effect was found for masked faces of elderly adult females, with smiling faces perceived as younger than neutral faces. This effect was of relatively small magnitude (less than one-year difference), but was evident in both experiments. Given its unpredicted direction, we can only speculate that this effect resulted from competing cues to age outside the covered region of the face (e.g. the forehead and neck), but we cannot provide a more specific mechanism beyond this speculation.

The similarity of the results, both in terms of the pattern of the AES in the different conditions, as well as the fact that the overall magnitude of the effect of smiling was similar for masked and unmasked faces, suggest that age is processed in a similar manner in the two conditions. An additional indication for shared processing of age in masked and unmasked faces is evident by the similar pattern of results in terms of average age judgments. In particular, the average perceived age across different combinations of age group and gender was similar for masked and unmasked faces and there was no indication for a general bias along the perceived age in masked faces.

This finding is at odds with the findings of recent studies that showed directional biases in age perception due to masks (Lau, 2021; Lau & Huckauf, 2021; Thorley et al., 2022). As we noted in the introduction, however, the contradictory biases could have resulted from item-specific effects due the small number of items used in these studies. Our results are also in odds with the results of another recent study that suggested that masked faces of middle-aged adults are perceived as younger than unmasked faces of the same individuals (Nicksic et al., 2021). As we noted previously in the introduction, this study suffered from substantial methodological problems preventing any firm conclusions to be made. For example, images used in that study were images of plastic surgery patients rather than of models from standard face databases. More importantly, lighting conditions were confounded with the manipulation of face masks due to unwarranted lighting artefacts reflected from the surface of the masks. Therefore, the uncovered parts of the faces in the masked and unmasked conditions contained different visual information. In the current study, we were able to avoid this pitfall by graphically superimposing masks on photographs of unmasked faces.

As for performance accuracy, there was some indication (in Experiment 2, but not in Experiment 1) for an effect of the mask on age evaluations. In other words, the average absolute error in age evaluations was larger in the masked condition (for similar pattern of results in age estimation accuracy see (Thorley et al., 2022) and in reaction times of age categorization, see (Fitousi et al., 2021)). We note that the magnitude of this effect can be considered as modest at best, with an average error of 7.24 vs. 6.85 years in the masked and the unmasked conditions, respectively. Still, the larger error in age evaluations for masked faces could result from a number of sources; first, it is possible that the lower part of the face, which includes the region of the mouth, carries additional age-related information about facial hair, wrinkling and pigmentation (Forte et al., 2015). In addition, given the importance of the overall shape of the face in face processing as well as in age evaluations (Roudaia et al., 2014), it is possible that the covering of parts of the face with a mask could interfere with the global processing of shape.

To summarize the results, we found that the smiling faces of young and middle-aged people appear older than neutral faces of the same individuals. This was true for both masked and unmasked faces, which suggests that the two are processed in a similar manner when one is evaluating age; i.e. wrinkling around the eyes, which increases when people smile, is a potent driver of age perception. Further evidence for this conclusion comes from the similar pattern of average age evaluations in masked and unmasked faces. The only notable difference in performance between the two face categories was a modest decrease in accuracy for masked faces, but such a decrease is expected when a part of the face is occluded with a mask. Overall, our results suggest that unlike other aspects of face processing, which are heavily impaired by masks (Carragher & Hancock, 2020; Freud et al., 2020; Grundmann et al., 2021), the evaluation of age in masked faces remains mostly intact. As we have already suggested, the fact that the perception of age is largely unaffected by the presence of a mask, is consistent with the idea that the perception of someone’s age is driven, at least in part, by wrinkles and other features that change with age in the upper part of the face.

## Data Availability

The dataset of results of the current study are available from the corresponding author upon request. The dataset will be deposited in a publicly available repository.
